# Animal modeling an oligodendrogliopathy – multiple system atrophy

**DOI:** 10.1186/s40478-016-0279-6

**Published:** 2016-02-09

**Authors:** Jonathan M. Bleasel, Glenda M. Halliday, Woojin Scott Kim

**Affiliations:** Neuroscience Research Australia, Barker St, Randwick, Sydney, NSW 2031 Australia; School of Medical Sciences, University of New South Wales, Sydney, NSW 2052 Australia

**Keywords:** Multiple system atrophy, α-Synuclein, Oligodendrocyte, Animal modeling

## Abstract

Multiple system atrophy (MSA) is a rare, yet rapidly-progressive neurodegenerative disease that presents clinically with autonomic failure in combination with parkinsonism or cerebellar ataxia. The definitive neuropathology differentiating MSA from Lewy body diseases is the presence of α-synuclein aggregates in oligodendrocytes (called glial cytoplasmic inclusion or GCI) rather than the fibrillar aggregates in neurons (called Lewy bodies). This makes the pathological pathway(s) in MSA unique in that oligodendrocytes are involved rather than predominantly neurons, as is most other neurodegenerative disorders. MSA is therefore regarded as an oligodendrogliopathy. The etiology of MSA is unknown. No definitive risk factors have been identified, although α-synuclein and other genes have been variably linked to MSA risk. Utilization of postmortem brain tissues has greatly advanced our understanding of GCI pathology and the subsequent neurodegeneration. However, extrapolating the early pathogenesis of MSA from such resource has been difficult and limiting. In recent years, cell and animal models developed for MSA have been instrumental in delineating unique MSA pathological pathways, as well as aiding in clinical phenotyping. The purpose of this review is to bring together and discuss various animal models that have been developed for MSA and how they have advanced our understanding of MSA pathogenesis, particularly the dynamics of α-synuclein aggregation. This review will also discuss how animal models have been used to explore potential therapeutic avenues for MSA, and future directions of MSA modeling.

## Introduction

**Fig. 1:**
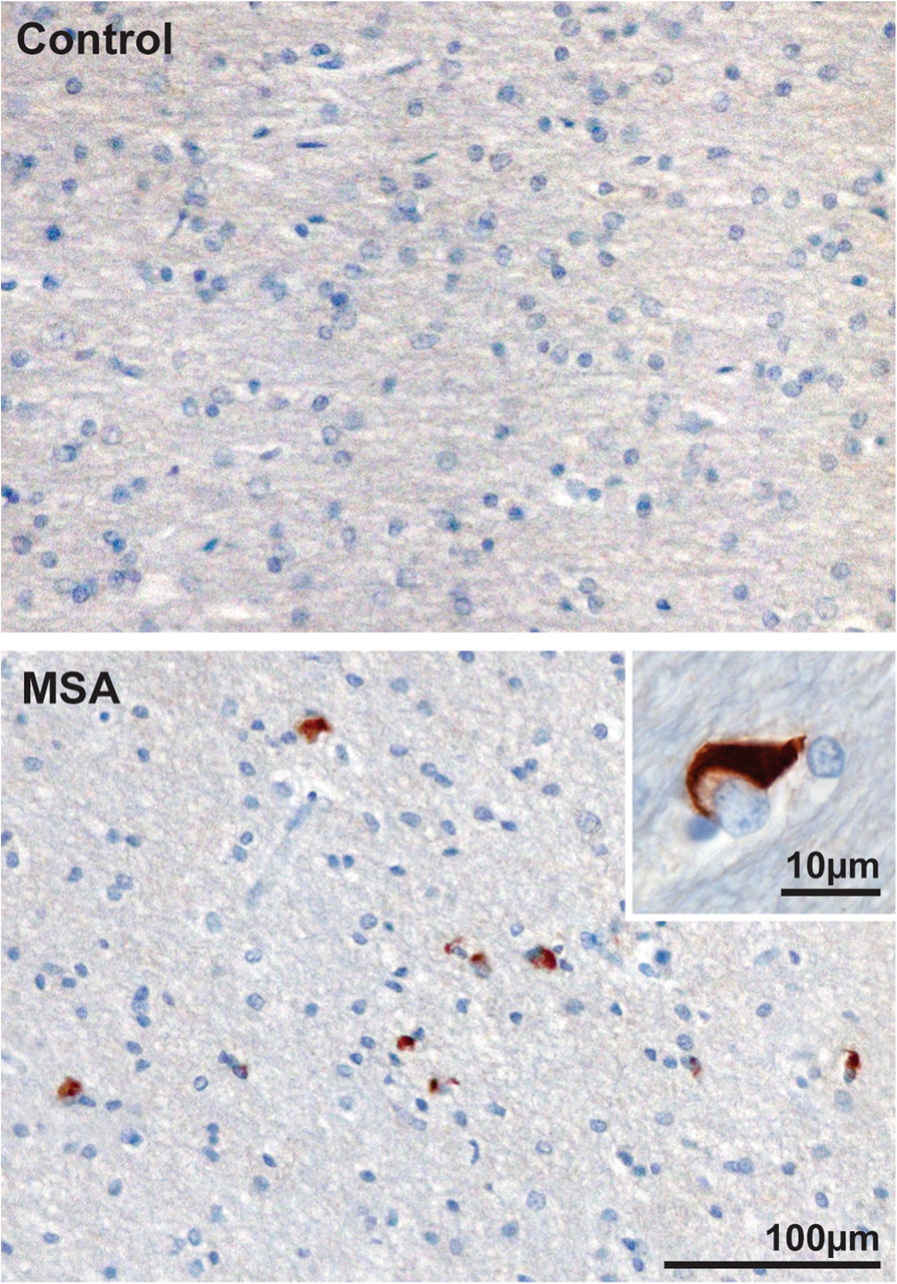
The pathological hallmark of MSA is the presence of glial cytoplasmic inclusions (GCIs) in the brain. Micrographs show white matter underlying the motor cortex stained using α-synuclein immunohistochemistry and Nissl counterstaining (0.5 % cresyl violet). The typical GCI aggregates in oligodendrocytes in MSA (also shown at higher power in inset) are not found in healthy controls

Multiple system atrophy is a fatal neurodegenerative disease characterized by the clinical triad of parkinsonism, cerebellar ataxia and autonomic disturbance. The annual incidence in the 50–99 year age group is 3 per 100,000 person years with a mean survival of 9 years from diagnosis [1, 2]. Underlying neuropathology is characterized by widespread proteinaceous inclusions in oligodendrocytes, the myelin producing support cells of the CNS. The glial cytoplasmic inclusions (GCIs) are accumulations of α-synuclein (Fig. 1), a protein enriched in synapses that also accumulates pathologically in neurons in Parkinson’s disease (PD) and dementia with Lewy bodies (DLB). GCIs and the resulting neurodegeneration occur in a typical multisystem distribution encompassing the striatonigral and olivopontocerebellar systems and autonomic nuclei of the brain stem and spinal cord [3, 4].

MSA can be divided into two main subtypes, a parkinsonian subtype (MSA-P) with striatonigral degeneration and a cerebellar subtype (MSA-C) with pontocerebellar degeneration [5]. Both subtypes feature autonomic dysfunction, with associated GCIs and degeneration in a distributed network of autonomic brain regions [5, 6]. Case series from different ethnic populations suggest a racial variation in the prevalence of MSA subtypes. In a UK cohort of 100 cases MSA-P predominated in 34 % while 17 % had MSA-C and 49 % had equally distributed pathology [6]. In contrast a Japanese cohort of 50 cases demonstrated MSA-C subtype in 40 %, equal pathology in 42 % and 18 % with MSA-P phenotype.

The etiology and early pathogenesis of MSA remain enigmatic. Epidemiological studies are limited by low case numbers and the difficulty of obtaining definitive clinical diagnosis [7–12]. Unlike PD, no single gene mutations linked to familial forms of the disease have been identified. Multiplex families with MSA have been reported [13–15], but no common genotype has been identified and the existence of true Mendelian forms of the disease is questionable. Candidate gene studies have found genetic variation in the *SNCA* locus associated with a risk of MSA [16–18]. However, these are not substantiated in all cohorts [12]. Furthermore a pioneering GWAS found no risk conferring loci on the *SNCA* gene [19]. Recently, two new genes, *COQ2* [20] and glucocerebrosidase (*GBA*) [21], have been identified as risk factors for MSA. Recent transcriptomic studies of MSA brain have provided new insights into the molecular pathology of MSA. RNA-Seq analyses have revealed alterations in a number of genes in MSA brain including alpha and beta hemoglobin (*HBA1*, *HBA2* and *HBB*) and transthyretin [22]. An analysis of microRNAs has revealed a widespread dysregulation of microRNAs in MSA brain [23]. Changes to microRNA-96 resulted in downregulation of the solute carrier protein family *SLC1A1* and *SLC6A6* [23]. Myelin instability is regarded as an early event in MSA pathogenesis and a recent study showed that the myelin lipids (sphingomyelin, sulfatide and galactosylceramide) were severely decreased in MSA white matter specifically in disease-affected regions, providing further clues to MSA pathogenesis [24, 25].

Despite the emergence of new data, in the absence of definitive genetic leads, understanding of early pathogenic events must be interpolated from studies of advanced cases that become available as postmortem specimens. For all these reasons the development of cell and animal models of MSA has been immensely important for our understanding of the disease. Unfortunately these same knowledge gaps, particularly lack of causative mutations, make generating valid models of MSA more difficult. Existing models rely on phenotypic replication of the major neuropathological feature of the disease, namely oligodendrocyte accumulation of α-synuclein. Nonetheless these models remain instrumental for exploring (1) the dynamics of α-synuclein aggregation in cells, (2) pathways from GCI formation to glial and neuronal degeneration, and (3) potential targets for neuroprotective and disease-modifying therapies. This review will describe animal models of MSA and discuss the insights gained as well as limitations and future directions of such research.

## Review

### Glial cytoplasmic inclusions

Substantial numbers of α-synuclein-positive GCIs are the distinguishing pathological hallmark of MSA. GCIs are typically located close to or surrounding the nucleus of oligodendrocytes (Fig. 1). Filamentous aggregations of phosphorylated α-synuclein, similar to GCIs, are also found in Schwann cells predominantly in the anterior nerve of the sacral cord [26]. GCIs have varied morphology ranging from triangular or conical to half-moon shaped [27]. Immunohistochemical studies have identified a growing list of proteins colocalized with α-synuclein in GCIs including p25α, αβ-crystallin, ubiquitin and tubulin [28]. Ultrastucturally GCIs are composed of loosely packed filaments of α-synuclein misfolded in a ß-sheet conformation [29, 30]. α-Synuclein in GCIs of MSA is phosphorylated at residue Ser-129, as is the case in Lewy bodies of PD and DLB [31]. Phosphorylated α-synuclein is also ubiquinated [32].

α-Synuclein is a cytosolic protein that occurs primarily in neurons where it has a propensity to associate with lipid membranes [33–36] and is concentrated in synapses. Characteristic changes in the solubility of α-synuclein are observed in homogenates of MSA-affected tissue. In the largest multi-region examination of α-synuclein solubility in MSA brains, Tong and colleagues [37] observed a dramatic accumulation of membrane associated α-synuclein (sodium dodecyl sulfate (SDS)-soluble fraction) specific to disease-affected regions, a finding confirmed in other studies [38–41]. These observations suggest a solubility shift of α-synuclein out of the cytosolic compartment may be a key step in MSA pathogenesis.

### Oligodendrocyte cell lines

Prior to developing animal models of MSA, in vitro studies have relied on the use of existing oligodendrocyte cell lines. Permanent oligodendrocyte cell lines include HOG [42] and KG1c [43], derived from resected human glioma specimens; MO3.13, adult human oligodendrocytes fused with a neoplastic cell line [44]; and OLN-93, derived from transformed rat oligodendrocytes [45]. The protein expression profiles and morphology of these cells bear resemblance to immature oligodendrocytes however some further in vitro differentiation can be induced in HOG and MO3.13 cells in which case cell proliferation is reduced [46]. Like primary human oligodendrocytes these clonal cell lines do not express significant levels of α-synuclein, thus genetic manipulation is required to reproduce the features of MSA.

**Fig. 2:**
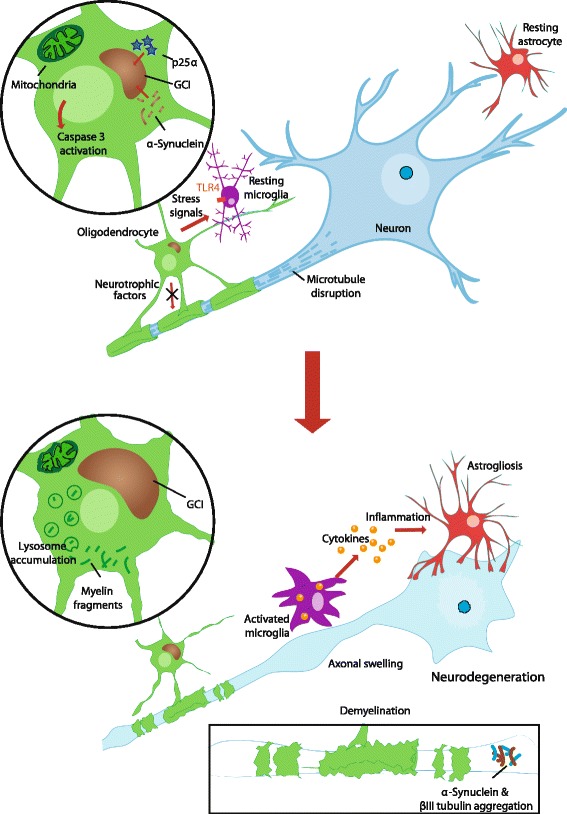
Insights into MSA pathophysiology. The sequence of pathological events of MSA is now recognized as abnormal protein aggregations (GCIs) in oligodendrocytes first, followed by demyelination and then neurodegeneration and loss of neurons. GCIs are predominantly composed of α-synuclein. Early features of MSA pathogenesis include decline in glial neurotrophic factors, mictrotubule disruption, and activation of caspase 3 and microglial toll-like receptor 4 (TLR4). Late features of MSA pathogenesis include accumulation of lysosomes and cytoplasmic myelin fragments in oligodendrocytes, increased production of cytokines from activated microglia, and astrogliosis

In an early cell-transduction study Stefanova and colleagues found that overexpression of α-synuclein in an astrocytoma cell line and rat primary mixed glial culture was sufficient to produce widespread fibrillar α-synuclein aggregates and evidence of cellular stress and degeneration [47]. Kragh and colleagues generated a successful cell model of MSA-like glial degeneration in OLN-93 cells by co-expressing α-synuclein and the oligodendrocyte microtubule-associated protein, p25α [48]. Cells expressing both proteins demonstrated α-synuclein aggregation and mictrotubule retraction in association with activation of the apoptotic protein caspase 3 (Fig. 2). These changes were absent in cells expressing either of the proteins alone or in combination with a host of control proteins including β-synuclein, CRMP-2 and Tau40. Alternative to in vitro transduction, primary cultures of oligodendrocytes and neurons can be prepared from the brains of transgenic rodent models of MSA [49]. While, not able to proliferate in vitro, these primary cultures provide an opportunity for closer examination of subcellular dynamics of α-synuclein accumulation and cytotoxicity in fully differentiated cells, as discussed below.

### Stereotaxic models

The earliest models of MSA reproduced L-dopa-unresponsive striatonigral degeneration (SND) through the use of neurotoxins, locally or systemically delivered. While of limited use in elucidating the pathogenic mechanisms underlying MSA, these models provided insights into the interactions between striatal and nigral lesions [50] and the neuropathological basis of SND symptomatology [50, 51]. Stereotaxic models have also been test beds for early non-dopaminergic therapies against the motor impairment of MSA, including embryonic neuronal grafts [52, 53]. Nonetheless, the most recent advances in modeling MSA have come from transgenic animal studies, which will be discussed in detail below.

### Transgenic murine models

#### Oligodendrocyte proteolipid protein promoter

Khale and colleagues first attempted to reproduce the features of MSA in mice expressing human α-synuclein (hα-syn) under an oligodendrocyte specific promoter, proteolipid protein promoter (PLP) [41]. The resulting transgenic mice (PLP-hα-syn) featured half-moon and triangle shaped α-synuclein-positive inclusions in oligodendrocytes similar to GCIs. These aggregates recapitulated other biochemical features of authentic GCIs including phosphorylated α-synuclein immunoreactivity and SDS detergent-insolubility. However no obvious demyelination or oligodendrocyte loss was observed in the transgenic or control mice aged up to 18 months (only cerebellar white matter tested). Similarly no neuronal loss or motor phenotype was discovered in mice up to 10 months old.

Later studies of the same mouse line found subtle but progressive motor impairment in mice after 12 months of age [54, 55]. Stefanova and colleagues [55] observed neuronal loss in the substantia nigra pars compacta (SNc) and locus coeruleus in PLP-hα-syn mice correlating with a subtle motor phenotype of reduced hind limb stride length. A more recent study [54] reported widespread α-synuclein pathology with distribution analysis revealing the highest burden in the globus pallidus. There was also a 31.4 % reduction in dopaminergic neurons in the SNc compared to non-transgenic mice. The motor phenotype was again subtle with progressive impairment in only one out of three tests.

The PLP-hα-syn mouse line has also shown some utility in replicating non-motor aspects of the MSA phenotype. Stemberger and colleagues [56] reported evidence of neurodegeneration in regions of the PLP-hα-syn mouse brain associated with autonomic failure and other non-motor symptoms, including the nucleus ambiguus in the brainstem and Onufs nucleus in the spinal cord. Kuzdas and colleagues [57] also observed neurodegeneration in the nucleus ambiguus and corresponding discrepancies in cardiac autonomic function compared to non-transgenic mice. Another study reported abnormal urodynamic studies and structural changes in the bladders of PLP-hα-syn mice suggestive of bladder dysfunction. This corresponded to neurodegeneration observed in micturition centers in the spinal cord and brainstem [58].

#### Oligodendrocyte myelin basic protein promoter

Shults et al. [59] produced a transgenic mouse line expressing α-synuclein in oligodendrocytes under the myelin basic protein promoter (MBP-hα-syn mice). Four lines were generated with varying levels of α-synuclein expression. Perinuclear α-synuclein inclusions with a fibrillar structure under electron microscopy were observed in oligodendrocytes. α-Synuclein in inclusions was hyperphosphorylated and ubiquitinated. The most extensive pathology was observed in regions commonly affected in MSA: the basal ganglia and cerebellum. MBP-hα-syn mice demonstrated loss of myelin staining and astrogliosis in white matter tracts. Ultrastuctural examination of affected oligodendrocytes revealed prominent mitochondrial abnormalities including enlarged and irregularly shaped organelles (Fig. 2). There were also extensive axonal alterations including decreased fiber density and irregular swelling of axons (Fig. 2). Dopaminergic neuronal loss in the SNc was demonstrated. The severity of both the GCI-like pathology and neurodegeneration positively correlated with level of α-synuclein expression between the different MBP-hα-syn lines. Motor phenotype severity also varied dramatically from severe tremor, ataxia, seizures and premature death in the highest expressing line to mild tremor and variable motor impairment in intermediate and lower expressing lines. Ubhi and colleagues reported olfactory deficits in MBP-hα-syn mice compared to non-transgenic controls using a buried pellet finding test. However, in contrast to PD, significant olfactory disturbance is not a consistent feature of MSA [60, 61].

#### 2’,3’-cyclic nucleotide 3’-phosphodiesterase promoter

The third MSA mouse model features expression of human α-synuclein under the 2’,3’-cyclic nucleotide 3’-phosphodiesterase promoter (CNP-hα-syn). Yazawa and colleagues [62] demonstrated age-related progressive motor deficits in CNP-hα-syn mice compared to age matched control oligodendrocytes. GCI-like α-synuclein inclusions and neurodegeneration were observed, most prominently in the cerebral cortex and spinal cord. There was a lack of overt degeneration observed in the cerebellum and pontine nuclei, associated with a lower level of α-synuclein expression in these regions. α-Synuclein solubility shifts were examined with serial extractions of homogenates from the spinal cord, cerebrum, cerebellum and brain stem. The pattern of high molecular weight insoluble α-synuclein in CNP-hα-syn mice was similar to analogous extractions from MSA tissue.

Ultrastructural examination of samples of cerebellum, pons, cerebrum and spinal cord was undertaken with immunocytochemistry assessed using transmission electromicroscopy. Oligodendrocytes from CNP-hα-syn mice demonstrated degraded myelin, extensive accumulation of lysosomes and cytoplasmic myelin fragments (Fig. 2), suggesting autophagocytosis of myelin. This was associated with α-synuclein positive filamentous inclusions in oligodendrocytes and accumulation of α-synuclein within the degenerating myelin sheath. Surprisingly while neuronal cell bodies were not immunostained for human α-synuclein in CNP-hα-syn mice, an antibody that recognizes endogenous mouse α-synuclein detected α-synuclein fibrils in a large number of axons in the spinal cord. Very little α-synuclein immunoreactivity was observed in axons of the spinal cord of control mice. This suggests that in this MSA model, α-synuclein-induced oligodendrocyte degeneration promotes accumulation of endogenous mouse α-synuclein in degenerating axons. α-Synuclein positive neuronal inclusions are a consistent feature of MSA histopathology, though their place in the pathogenic sequence has remained enigmatic [63]. A recent study highlighted the neuronal accumulation of α-synuclein in MSA [64], although the distribution and variety of such inclusions were not observed using previous silver stains [65] and do not associate with the pattern of neuronal loss observed, suggesting such α-synuclein accumulations are not of the same toxic conformation as those found in GCIs [66].

### Determining the pathogenesis of MSA

The recapitulation of GCI-like inclusions in animal and cell models of MSA offers a valuable opportunity to study the late intracellular pathological events, including the dynamics of α-synuclein aggregation, determinants of α-synuclein-mediated toxicity and mechanisms of secondary neurodegeneration (Fig. 2).

#### α-Synuclein interaction with microtubules

The association of α-synuclein with microtubule related proteins is of great interest to the physiological and pathological functions of the protein (Fig. 2). In cultured neurons, α-synuclein appears to regulate the expression and localization of norepinephrine transporter in a manner dependent on interaction with the microtubule cytoskeleton (treatment with depolymerizing agent nocodazole abolished regulatory activity) [67]. Furthermore, microtubule-dependent trafficking was impaired in cells overexpressing α-synuclein [68]. A number of microtubule-interacting proteins have been implicated in α-synuclein aggregation or are found colocalized with α-synuclein in pathological aggregates in both MSA and PD, including tubulin [69], p25α [70] and tau [71].

In the OLN-93 model of MSA generated by Kragh and colleagues [48], MSA-like glial degeneration was induced specifically by co-expression of α-synuclein with p25α, an oligodendrocyte protein involved in basic microtubule assembly and myelination [72, 73]. Varying the levels of α-synuclein expression further showed that there was a low threshold for cytotoxicity when p25α was present. Apart from being colocalized with α-synuclein in GCIs, p25α promotes the fibrilization of α-synuclein in vitro [70]. Moreover, Song and colleagues [74] observed relocalization of p25α from the myelin sheath to the swollen cell soma in MSA oligodendrocytes before the appearance of α-synuclein in GCIs. A recent study showed that p25α was relocated from the nucleus to the perinuclear cytoplasm in the oligodendrocytes of sporadic and COQ2 MSA [75]. These results and evidence from the cell model support the notion that microtubule disruption, possibly linked to dysfunctional myelination, is a fundamental event in MSA pathogenesis.

In degenerating neurons from the CNP-hα-syn mouse, a neuronal microtubule element, β-III tubulin, was observed to be colocalized with endogenous α-synuclein aggregates [49]. A more recent study [76] further examined the role of β-III tubulin association in the secondary neuronal pathology using the same model of CNP-hα-syn oligodendrocyte/neuron primary cultures. The microtubule depolymerizing agent nocodazole significantly reduced α-synuclein aggregation in the transgenic cells while rifampicin, a putative inhibitor of α-synuclein self-association, had no effect. This suggests that in this instance, association of α-synuclein with microtubule components may be even more important than self-association of the protein in its pathological aggregation. Ito and colleges [77] studied the electrophysiological consequences of endogenous α-synuclein accumulation in neurons in the brains of CNP-hα-syn mice. Miniature excitatory and inhibitory post-synaptic currents (mEPSC/mIPSC) were measured in freshly dissected mouse brain tissue; results showed a significant decrease in mIPSC in transgenic mice compared to control. Again, treatment with microtubule depolymerizing agent nocodazole rescued GABAergic dysfunction.

In summary α-synuclein function and pathology is closely connected with the microtubule system in both oligodendrocytes and neurons (Fig. 2). Further understanding of these associations may elucidate the early pathogenic events occurring in oligodendrocytes prior to GCI formation as well as providing potential therapeutic targets to ameliorate α-synuclein accumulation and toxicity.

#### Increased susceptibility to oxidative stress

As shown in the early stereotaxic models of SND, neurons in the striatum and SNc are uniquely susceptible to oxidative stress induced by the mitochondrial toxins 3-NP and MPTP, respectively [78]. The link between oxidative stress and pathogenesis of both PD and MSA has been explored in many experimental and epidemiological studies. The animal and cell models of MSA described above have yielded particularly intriguing results regarding susceptibility to and impacts of oxidative stress in oligodendrocytes overexpressing α-synuclein.

In animal models of MSA, intoxication with 3-NP has the effect of enhancing the phenotype of MBP-hα-syn and PLP-hα-syn mice to more closely resemble human disease. In the PLP-hα-syn mouse, known for a relatively benign motor and neurodegenerative phenotype, 3-NP treatment caused a significant deterioration in motor behavior, which was greater than that in non-transgenic mice undergoing the same regimen of intoxication. Histopathologically 3-NP produced neurodegeneration in typical MSA regions; the cerebellum, pons and inferior olive. 3-NP did not appear to augment α-synuclein pathology in transgenic or control mice. Intoxication of MBP-hα-syn mice with 3-NP [79] intensified neuropathology and motor deficits compared to vehicle treated transgenic mice and 3-NP and vehicle treated non-transgenic mice. In terms of regional selectivity, 3-NP administration appeared to exacerbate neuronal and oligodendrocyte pathology in most regions associated with MSA, including the SNc and striatum. However, oligodendrocyte loss in cerebellar white matter was not significantly exacerbated by 3-NP. These results are consistent with an increased vulnerability of α-synuclein overexpressing oligodendrocytes to oxidative stress and a regional selectivity of this effect that corresponds to the distribution of MSA-P pathology.

3-NP intoxication of MBP-hα-syn mice also resulted in biochemical changes resembling those reported in human disease [79]. These included post-translational modifications of α-synuclein, with greater expression of nitrated α-synuclein detected in oligodendrocytes of 3-NP treated MBP-hα-syn mice compared to other groups. Analysis of fractions of α-synuclein from brain homogenates extracted with different detergents revealed greater quantity of oxidated and nitrated α-synuclein in less soluble fractions of transgenic mice brains treated with 3-NP. Conversely, in the more soluble fraction, 3-NP treatment reduced nitrated α-synuclein in MBP-hα-syn mice brain compared to vehicle treated MBP-hα-syn mice. This suggests that oxidative stress and resulting post-translational modifications of α-synuclein may play a role in the solubility shifts noted in MSA brain tissue [37].

#### Proteolytic dysfunction

As a major pathway for intracellular protein degradation, the ubiquitin-proteasomal system has been a focus of interest in neurodegenerative conditions featuring protein misfolding and aggregation. Decreased proteasomal activity and/or down-regulation of proteasomal subunits has been observed in several such conditions including Alzheimer’s disease [80], DLB [81], PD [82], progressive supranuclear palsy and MSA [83].

In the PLP-hα-syn model of MSA induction of proteolytic stress by systemic proteasomal inhibition (PSI) resulted in a remarkable exacerbation of the motor and neurodegenerative phenotype closely resembling human disease [84]. Most notably PSI treatment resulted in significant neurodegeneration in the SNc, striatum, cerebellar cortex (Purkinje fibres), inferior olive and pontine nuclei but not regions typically spared by MSA, the nucleus accumbens and deep cerebellar nuclei [84]. The same regimen of PSI treatment had no discernible effect on non-transgenic mice. Histopathologically, more severe myelin pathology (thinning of myelin sheaths or dilation and occupation with fibrillary material) and oligodendrocyte degeneration were observed in affected regions of PSI treated PLP-hα-syn mice compared to vehicle-treated littermates [84]. There was also an approximately 25 % increase in α-synuclein immunoreactivity in PSI-treated compared to vehicle treated transgenic mice. Large fibrillar hα-syn inclusions were observed in the myelin sheath after PSI-treatment, which were rarely seen in vehicle treated PLP-hα-syn mice [84].

Proteasomal stress was also found to cause MSA-like pathology in a cell model featuring overexpression of α-synuclein in OLN-93 oligodendrocytes without p25α coexpression. Riedel and colleagues [85] found no fibrillary inclusions or cytotoxicity in OLN-93 cells co-expressing α-synuclein and tau. However, treatment with either hydrogen peroxide (oxidative stress) or the proteasomal inhibitor MG-132 caused recruitment of tau to α-synuclein-positive aggregates and the formation of filamentous structures. Treatment with both stressors caused a shift of α-synuclein to more insoluble forms, mimicking the change in solubility seen in MSA specimens.

The extent to which proteasomal inhibition enhances MSA-like pathology in these models of oligodendrocyte α-synuclein overexpression strongly suggests that proteasomal dysfunction plays an important role in the pathophysiology of MSA. Further insights into the mechanism of proteasomal dysfunction may open the way for therapeutic targets to ameliorate α-synuclein cytotoxicity.

#### Loss of oligodendrocyte trophic support

Loss of trophic support from oligodendrocytes in MSA has been postulated as the mechanism for neurodegeneration secondary to the primary glial pathology [86–88]. Ubhi et al. [89] investigated the differential expression of neurotrophic factors in MBP-hα-syn mice and non-transgenic control oligodendrocytes. MBP-hα-syn mice were compared with two lines overexpressing α-synuclein in neurons (PDGFβ-hα-syn and mThy1-hα-syn), α-synuclein knockout mice and non-transgenic control oligodendrocytes. Out of three putatively glial-derived neurotrophic factors, glial-cell derived neurotrophic factor (GDNF), brain derived neurotrophic factor (BDNF) and insulin like growth factor 1 (IGF-1), only GDNF was specifically decreased in the MBP-hα-syn mice. In situ hybridization revealed no difference in GDNF mRNA suggesting that α-synuclein overexpression exerts a post-translational effect on its expression. Immunoblot of two MSA-affected regions from a small sample of MSA (*n* = 3) and non-neurodegenerative control brains (*n* = 3) suggested that reduction in GDNF expression is also a feature of human disease. The authors proceeded to investigate the neuroprotective efficacy of GDNF infusions in MBP-hα-syn mice, which is discussed below with other experimental therapies. This study suggests that the specific decline in this glial neurotrophic factor as a result of GCI-like pathology may be responsible for the neurodegeneration and clinical phenotype previously reported in the MBP-hα-syn mouse [59].

The CNP-hα-syn mouse has also proved a useful model for investigating the pathway from primary oligodendrocyte pathology to neurodegeneration. As described above, Yazawa and colleagues [62] observed a surprising accumulation of endogenous mouse α-synuclein in neurons in response to oligodendrocyte overexpression of the human form of the protein in CNP-hα-syn mice. Nakayama and colleagues [49] replicated this neuronal accumulation of endogenous α-synuclein in primary oligodendrocyte/neuron cultures derived from CNP-hα-syn mice. The authors hypothesized that neuronal α-synuclein accumulation depended on oligodendrocyte dysfunction caused by CNP-hα-syn overexpression. They found that arresting glial proliferation in these cultures by treatment with cytosine-1-β-D-arabinofuranoside ameliorated the neuronal α-synuclein accumulation. Furthermore treatment of wild type oligodendrocyte/neuron cultures with conditioned media from CNP-hα-syn cells resulted in accumulation of endogenous α-synuclein in the previously unaffected non-transgenic cells. These results suggest that pathological signals from degenerating oligodendrocytes transmitted to myelinated axons and into the extra-cellular environment may alter neuronal α-synuclein expression and contribute to neurodegeneration.

#### Prion-like transmission of α-synuclein

Although the above investigations lend support to the categorization of MSA as a primary oligodendrogliopathy with secondary neurodegeneration, a number of investigators have studied the potential role of neuron to glia transmission of α-synuclein in animal and cell models. The prion-like transmission of α-synuclein has long been of interest as both a mode of propagation of pathology in PD and as an explanation for the accumulation of this predominantly neuronal protein in oligodendrocytes in MSA. α-Synuclein exocytosis from neurons has been well documented [90, 91] and various forms of the protein can be found in cerebrospinal fluid suggesting a certain amount of physiological secretion [92, 93]. Uptake of extracellular α-synuclein by neurons has also been documented resulting in aggregate formation and cell death in some studies [94–96]. In vivo, the transmission of α-synuclein from neuron to neuron is suggested by appearance of α-synuclein pathology in tissue grafts (containing dopaminergic neuroblasts) in PD brains many years after transplantation [97, 98]. Uptake by other cell types, astrocytes and microglia, has been documented both from a culture medium and the secretion of neurons in co-culture, causing an inflammatory response in the case of astrocytes [95, 99].

More recent studies have shown that oligodendrocytes also take up monomer and oligomer forms of α-synuclein into their cytosol [100–102], apparently via dynamin-depednent endocytosis [101, 102]. In KG1C oligodendrocytes, the recombinant α-synuclein taken up by the cells formed aggregates of varying size, including some larger perinuclear inclusions [101]. These had several features of authentic GCIs including thioflavin S staining indicative of fibrillar structure, ubiquitination, phosphorylation at S129 and colocalization with p25α. Uptake of α-synuclein was also observed when KG1C cells were cultured with SH-SY5Y neurons overexpressing α-synuclein. Reyes and colleagues [102] injected fluorescent-labeled α-synuclein into the neocortex of mice. Slices examined only 1 hour after injection showed monomer and oligomer forms of α-synuclein had entered oligodendrocytes around the injection site and were present in a diffuse or punctate pattern throughout the cytoplasm. In another study, Watts and colleagues injected brain homogenates of MSA patients into the brains of mice that had no propensity to develop neurological disease [103]. Inoculation with MSA homogenates induced a rapidly progressive neurological disease characterized histologically by widespread neuronal α-synuclein deposition; however, uptake by oligodendrocytes in particular was not reported. Mice inoculated with homogenate from age matched non-neurodegenerative brains showed no such changes. In a follow-up study Prusiner and colleagues demonstrated that brain extracts from 14 MSA cases all transmitted neurodegeneration to mice with development of neuronal α-synuclein deposition [104]. In contrast, brain extracts from PD and control cases did not induce aggregation of α-synuclein, suggesting that the strain of α-synuclein found in MSA brain is different to that of PD brain [104].

Cases of neuron to oligodendrocyte α-synuclein transmission in vivo have also been reported. Reyes and colleagues [102] examined mice that had been injected with a viral vector encoding human α-synuclein [105]. Three weeks later embryonic rat tissue from the ventral mesencephalon was transplanted into the region of the transduction. The authors found that in addition to α-synuclein transmission to neurons in the grafted tissue, oligodendrocyte precursor cells and mature oligodendrocytes present in the transplant also demonstrated human α-synuclein immunopositivity. Studies in a double transgenic mouse model (oligodendrocyte MBP-hα-syn and neuron PDGF-hα-syn) demonstrated that α-synuclein accumulation redistributed in oligodendrocytes comparable to the single transgenic MBP-hα-syn model [106]. The authors provided three possible causes for this phenomenon – redistribution of α-synuclein from neurons to oligodendrocytes; suppression of neuronal expression by unknown signals from oligodendrocytes; and neuronal clearance of α-synuclein.

Thus the prion-like hypothesis of α-synuclein transmission in MSA has gained some support from both in vitro and in vivo studies. These models of oligodendrocyte α-synuclein accumulation dispense with the need for transcriptional upregulation of the protein and are thus more consistent with the current evidence from human studies of MSA brain tissue [107–109]. However while potentially more valid on the basis of known pathogenesis, none of these models have so far achieved the phenotypic resemblance to true MSA described in the main transgenic animal models.

### Exploring therapies for MSA

Animal and cell models provide essential platforms for preclinical trials of prospective neuroprotective and disease modifying therapies in MSA.

#### Anti-inflammatory therapy

Astrogliosis and microglial activation are prominent neuropathological findings in many of the animal models described above [55, 59, 62, 110]. Stefanova and colleagues [110] observed that microglial activation and toll-like receptor 4 (TLR4) expression was positively correlated with neurodegeneration in the PLP-hα-syn mouse (Fig. 2). They proceeded to investigate the effect of treatment with minocycline, a microglial activation inhibitor, in PLP-hα-syn mice from 2 to 4 months of age (before the earliest observed onset of neuronal loss in the SNc). Early minocycline treatment resulted in a significant reduction in iNOS and TLR4 expression and ameliorated neuronal loss in the SNc compared to vehicle-treated transgenic mice.

Similarly myeloperoxidase (MPO) has been targeted due to its role in immune cell-mediated oxidative damage in MSA and other neurodegenerative diseases. Treatment of 6-month old PLP-hα-syn mice intoxicated by 3-NP with an MPO inhibitor (MPO-I) resulted in a dose dependent improvement in motor deficits and protection of neurons in the striatum and SNc [111]. These results were accompanied by reduced markers of inflammation in the affected regions of MPO-I treated mice compared to vehicle treated control oligodendrocytes. Interestingly the authors also showed a reduction of GCI-like pathology in MPO-I treated mice and reduced prominence of the nitrated α-synuclein species previously noted in this animal model [79]. These results suggest that inflammation plays an important role in neurodegeneration in MSA (Fig. 2) and may even have a more fundamental influence on α-synuclein modification and GCI formation. However, these studies also show that early intervention with anti-inflammatory agents, preferably before onset of overt neuropathology, is necessary to achieve significant neuroprotection [110, 112]. This may limit the utility of such therapies in human disease where neuropathology is almost always well established by the time of diagnosis. Nonetheless, further investigation of anti-neuroinflammatory therapies in MSA is certainly warranted.

#### Neurotrophic factor augmentation

Rasagiline is an inhibitor of monoamine oxidase type B (MAOB) whose use as a symptomatic treatment in PD is well established [113, 114]. In vitro studies have also suggested a neuroprotective effect in PD models, though human trials have been less convincing [115]. The neuroprotective potential of rasagiline has been linked to induction of GDNF [116, 117], an oligodendrocyte-derived factor that was selectively reduced in the brains of MBP-hα-syn mice as described above [89]. Infusion of GDNF itself has been proposed as a potential neuroprotective therapy and was found to ameliorate clinical deficits and dendritic pathology (loss of MAP2 immunoreactive neruopil) in MBP-hα-syn mice [89]. Thus, if its purported mechanism is legitimate, rasagiline may offer a less invasive alternative for augmentation of GDNF in MSA brains. In the PLP-hα-syn mouse model augmented with 3-NP intoxication, Stefanova and colleagues [118] tested the neuroprotective efficacy of low (0.8 mg/kg daily) and high-dose (2.5 mg/kg) rasagiline in mice aged over 6 months. High does rasagiline (comparable to a reportedly tolerable human dose of 12 mg/day) was associated with improvement in motor performance indicators compared to control treated transgenic mice. On neuropathological examination, high dose rasagiline mitigated neuronal loss in the striatum, cerebellar cortex, inferior olive, SNc and pontine nuclei. This improvement was dramatic in the SNc and pontine nuclei with neuron levels comparable to healthy controls despite 30-60 % oligodendrocyte loss in saline-treated transgenic mice.

#### Mesenchymal stem cells

The use of tissue grafts has long been of interest in neurodegenerative diseases, particularly those with relatively focal lesions such as PD. Early stereotaxic models of MSA were frequently used to evaluate the efficacy of grafting neural tissue into toxin-ablated basal ganglia. More recently, however, the use of mesenchymal stem cells (MSC) has presented itself as a relatively less invasive and autologous procedure for regenerating damaged neural pathways. Park and colleagues [119] found that a single dose of human mesenchymal stem cells ameliorated neuron loss and motor deficit in a double-toxin mouse model comprising concurrent administration of MPTP and 3-NP to induce nigral and striatal degeneration respectively. Rescue of neurons in these regions correlated with significantly reduced astro- and micro-gliosis compared with saline treated intoxicated animals, suggesting a possible immunomodulatory effect of the MSC.

The effect of MSC infusion has also been tested in the transgenic PLP-hα-syn mice [120]. PLP-hα-syn mice aged 18 months (*n* = 12) were infused with mouse MSC. Treatment did not appear to be associated with improvements in motor behavior compared to saline treated transgenic mice (*n* = 6). However no healthy non-transgenic controls were tested to assess the extent of the baseline functional deficit in the PLP-hα-syn mice, which has been noted to have a relatively subtle motor phenotype. Nonetheless there did appear to be a modest but significant recovery of dopamine neurons in the SNc of the MSC group 4 weeks post treatment. α-Synuclein levels in midbrain-brainstem lysates were not altered between the two groups. A range of inflammatory cytokines in these lysates was also tested and a significant down regulation was observed for IL1α, IL-2, IL-10, IL-17, GM-CSF, TGF-β1 and TNF-α. The reduction of some of these cytokines was significantly correlated to extent of neuronal rescue in the MSC-treated mice. Although human MSC in the double toxin study were identified histologically in the brains of treated mice, the survival and fate of the transplanted cells in the transgenic mice was less certain. It will be difficult to determine whether the mechanism by which transplanted MSC provide benefit is through in vivo differentiation to replace damaged cells or via indirect immunomodulatory and trophic effects [121, 122].

#### Erythropoietin

Erythropoietin has been found to play a role in neuroprotection and repair after ischaemic and hypoxic injury in the brain [123, 124]. In vitro, erythropoietin promotes differentiation and survival of dopamine neurons in environments with differing oxygen tension [125]. A neuroprotective effect was also found in vivo with protection against MTPT toxicity in mice [126] and survival of dopamine neurons in mesencephalic grafts in a rodent model of PD [127]. Building on this evidence, Kollensperger and colleagues [128] examined the disease modifying potential of peripherally delivered erythropoietin in the PLP-hα-synuclein mouse model augmented with chronic 3-NP intoxication. PLP-hα-syn animals intoxicated with 3-NP were treated with either saline, early erythropoietin (before intoxication) or late erythropoietin (after intoxication). Unintoxicated transgenic mice treated with erythropoietin were used as control. The results demonstrated a protective effect of erythropoietin in both behavioral tests and neuronal loss in both the SNc and the striatum. The study suggests erythropoietin mitigates the enhanced toxicity of 3-NP usually observed in this model of oligodendrocyte α-synuclein overexpression. The specificity of protection against 3-NP toxicity in this model could not be determined due to the lack of a non-erythropoietin non-intoxicated control group. The prominent non-neurological actions of erythropoietin, particularly in hematopoiesis, make it an uncertain prospect for long-term trials in humans.

#### Targeting GCI formation

Finally, models of MSA that recapitulate α-synuclein aggregation and toxicity provide a platform for testing therapies that aim to interrupt or reverse this key pathogenic event. In the α-synuclein/p25α expressing OLN-93 model, Kragh and colleagues [48] tested three different inhibitors of α-synuclein aggregation (Congo Red, ASI1D peptide, and baicalein). All these agents significantly attenuated the microtubule retraction and apoptotic protein activation seen in untreated cells.

As noted above both rifampicin (a putative inhibitor of α-synuclein self-association) and nocodazole (microtubule depolymerizing agent) have been tested in primary cultures of oligodendrocytes and neurons from CNP-hα-syn mice. Daily intraperitoneal rifampicin injections also showed promising results in MBP-hα-syn mice (average 12 month old) [129]. Rifampicin treatment significantly reduced α-synuclein aggregates, loss of dendritic complexity, neuronal loss and astrogliosis compared to saline treated transgenic mice. Dendritic complexity (MAP2 immunoreactivity) and neuronal cell counts in rifampicin treated MBP-hα-syn mice were comparable to healthy vehicle-treated non-transgenic control oligodendrocytes. A recent double-blinded placebo-controlled trial of rifampicin in 100 MSA patients over 12 months did not show any effect on disease progression as measured by a clinical rating scale [130]. Despite some evidence of anti-aggregating properties in vitro, nocodazole is not a strong candidate for animal and human trials due to its unacceptable side effect profile.

### Limitations of current models and future directions

#### Validity of α-synuclein overexpression

Despite the success of these models in replicating features of the disease downstream of α-synuclein aggregation, it remains a difficult task to reconcile this simulated mechanism with evidence from extensive studies of MSA brain tissue. For one, demonstration of α-synuclein upregulation at the transcriptional level has been stubbornly allusive in human postmortem studies [107–109]. Indeed, the level of physiological α-synuclein expression in oligodendrocytes is questionable with only a few studies demonstrating expression in this cell population under normal conditions [131–133]. In contrast, duplication and triplication of the *SNCA* locus has been associated with both familial and sporadic forms of PD [134–136]. Three point mutations of the *SNCA* locus are also associated with familial PD. Thus the status of α-synuclein as the likely causative pathogenic molecule in PD does not necessarily hold true in MSA. Nonetheless, primary or secondary upregulation of α-synuclein in oligodendrocytes may yet be implicated in MSA as there is scope for more sensitive and cell-specific assays of oligodendrocyte expression patterns in MSA specimens.

#### Distribution of pathology

The classical distribution of glial and neuronal degeneration in human disease has been the most difficult aspect to replicate in the three basic murine models of MSA. Initial studies of the PLP-hα-syn mouse found no obvious neurodegeneration [41], while later studies suggested GCI formation and neural loss predominating in the basal ganglia, particularly the SNc [54, 55]. CNP-hα-syn mice demonstrated most significant pathology in the spinal cord, while MBP-hα-syn mice showed the most classical distribution with both the basal ganglia and cerebellum severely affected. The reasons for these regional disparities between mouse lines sharing the same basic defect are obscure. When Shults and colleagues [59] generated distinct MBP-hα-syn lines with differing levels of α-synuclein expression, there was a dramatic clinical and neuropathological spectrum between the lowest and highest expressing lines. Thus the disparate neurodegenerative phenotypes between transgenic lines could result from different efficiencies of the promoters expressing α-synuclein. However, the particular disparities in distribution of pathology may also suggest that the three promoters target different subtypes of oligodendrocytes.

The disparities in regional distribution of pathology in MSA mouse models may shed light on the existence of the subtypes MSA-C and MSA-P in human disease. The transgenic mouse lines described above do not reproduce the specific predominance of SND or olivopontocerebellar atrophy. However, investigation of factors that modulate the regional specificity of α-synuclein-mediated pathology, such as 3-NP intoxication and proteasomal inhibition, could shed light on the mechanism underlying the different phenotypes.

To this end, more accurate characterization of the three transgenic mouse lines is required. In particular, a quantitative investigation of regional differences in the level of oligodendrocyte α-synuclein expression and the extent of GCI formation and neurodegeneration is required. It is important that control regions not typically associated with MSA be included in these assays to test the specificity of neuropathology in each models. A quantitative comparison of α-synuclein expression levels between models may determine whether the extent and distribution of neuropathology is correlated with different efficiency of the three promoters.

#### Induced pluripotent stem cells

**Fig. 3:**
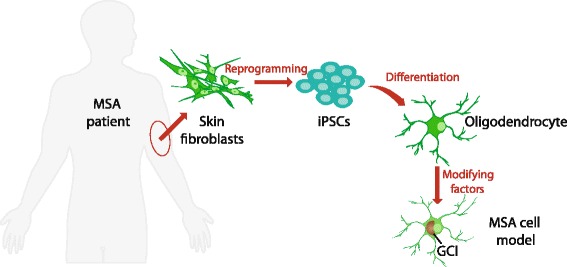
Development of induced pluripotent stem cells (iPSCs) from MSA patients represents the most advanced in vitro modeling of MSA. Somatic cells, such as skin fibroblasts, are reprogrammed into iPSCs using specific transcription factors. iPSCs are then differentiated into oligodendrocytes that feature the key phenotype – glial cytoplasmic inclusion (GCI)

While the cell models described above have proved useful in furthering understanding of MSA pathology, there are a number of disadvantages to the common cell lines in use. As described above, common permanent oligodendrocyte cell lines have undergone some manner of genetic modification to drive immortal cell growth and may carry other artifacts to tissue culture [137]. While the use of primary human cells would mitigate these pitfalls, they are understandably difficult to obtain and do not propagate well in culture. Pluripotent stem cells such as those obtained from early embryos are able to proliferate in vitro and differentiate into almost any mature cell type. Somatic cells isolated from adult patients (in most cases from skin or bone marrow biopsies) can be reprogrammed into a pluripotent state by overexpressing four transcription factors (Oct4, Sox2, Klf4 and c-Myc) [138]. The resulting cells are termed induced pluripotent stem cells (iPSCs) (Fig. 3). iPSCs can thus be derived from patients with specific genetic or sporadic diseases. Patient-specific iPSCs are then differentiated to the key cell types that are affected in the disease in question.

Pluripotent stem cells have been successfully derived from patients with neurodegenerative diseases and have yielded insights into pathophysiology and therapeutic approaches. The first human iPSCs shown to model the specific pathology seen in a genetically inherited disease were derived from a child with spinal muscular atrophy [139]. iPSC derived from four patients with Machado-Joseph disease (Spinocerebellar ataxia type 3) were differentiated into neurons and displayed a phenotype of insoluble protein aggregate formation [140]. The authors used these cells to demonstrate the key proteolysis of ataxin 3 that precipitated aggregate formation. Differentiation of the same iPSCs into glia allowed the authors to show the specific vulnerability of neurons to the disease process.

Recently, iPSCs were successfully derived from PD [141–143] and MSA [144] patients and were differentiated into neurons and oligodendrocytes respectively. As a disease whose etiology is almost certainly multi-factorial, MSA-derived iPSCs are likely to require additional modifying factors to generate a phenotype (Fig. 3). Factors that accelerate pathology such as oxidative stress may still need to be used to manifest the disease phenotype. Nonetheless MSA-specific iPSC could be the key to solving some of the enigmas surrounding this disease, including what precedes α-synuclein aggregation in disease-affected oligodendrocytes, the role of transcriptional upregulation of α-synuclein and the mechanism of neurodegeneration following oligodendrocyte demise.

### Conclusion

MSA is a devastating disease with extremely poor prognosis. The cause of MSA is unknown and there is no cure and current treatments are directed to addressing only the symptoms. Recent studies utilizing animal models that replicate the dynamics of α-synuclein aggregation in oligodendrocytes and the subsequent neurodegeneration have made significant inroads in our understanding of MSA pathogenesis. Such models have been instrumental in determining the sequence of pathogenic events in MSA brain, in particular the early events leading up to GCI formation. They have helped to identify molecular targets that could be exploited to inhibit α-synuclein deposition in oligodendrocytes, providing potential therapeutic avenues for MSA therapy. New developments in iPSC modeling will provide further insights into the disease mechanisms underlying this oligodendrogliopathy.
